# The use of *Caralluma fimbriata* as an appetite suppressant and weight loss supplement: a systematic review and meta-analysis of clinical trials

**DOI:** 10.1186/s12906-021-03450-8

**Published:** 2021-11-10

**Authors:** Ranil Jayawardena, Tormalli V. Francis, Sachith Abhayaratna, Priyanga Ranasinghe

**Affiliations:** 1 Department of Physiology, Faculty of Medicine, University of Colombo, Colombo, Sri Lanka; 2 Institute of Health and Biomedical Innovation, Queensland University of Technology, Brisbane, Australia; 3 Health and Wellness Unit, Faculty of Medicine, University of Colombo, Colombo, Sri Lanka; 4 Department of Pharmacology, Faculty of Medicine, University of Colombo, Colombo, Sri Lanka

**Keywords:** *Caralluma fimbriata*, Obesity, Appetite, Weight loss, Slimluma

## Abstract

**Background:**

Obesity prevalence has increased during the past few decades, causing a pandemic with an influx in other co-morbidities. Many factors influence weight gain in an obesogenic environment therefore strategies for treating obesity may vary from conventional dietary and physical activity interventions to pharamacotherapy. A shift in unconventional strategies as herbal products for treating obesity have been investigated and one such plant extract is *Caralluma fimbriata* (*C. fimbriata*). Further, the studies included were systematically reviewed to gather evidence on potential effects of *C. fimbriata* as an appetite suppressant and weight loss supplement.

**Methods:**

A systematic review of clinical trials reporting the effects of *C. fimbriata* as appetite suppression and anti-obesity supplement was reported according to PRISMA guidelines. Data were obtained by searching three databases: PubMed®, Web of Science® and SciVerse Scopus® for studies published until 30th April 2020.

**Results:**

A total of 7 articles studying *C. fimbriata* satisfied the inclusion and exclusion criteria and were sourced from various countries including Australia (3), Cuba (1), India (2) and Spain (1). Almost all studies recruited adults who were overweight or obese with a BMI > 25 kg/m^2^ (*n* = 5), with the exception of two studies, one that recruited healthy adults with a BMI average of 26.5 kg/m2 and the second one utilised a population of children and adolescents with Prader-Willis Syndrome (PWS). Parameters assessing obesity, biochemical and appetite factors were analysed by carrying out a meta-analysis. Compared to placebo controlled group, *C. fimbriata* extract significantly reduced WC by 1.59 cm (95% CI, − 3.07 to − 0.10, *p* = 0.041) and WHR by 0.06 (95% CI, − 0.12 to − 0.01, *p* = 0.05) although no significant effects were seen on BW, BMI and HC. Biochemical and appetite parameters outcome on *C. fimbriata* consumption had no significant changes. Any side effects of individuals who ingested the extract were reported by few studies of which most common effects were constipation, diarrhoea, nausea and rashes.

**Conclusion:**

Appetite parameters showed no significant changes and metabolic parameters did not improve with *C.fimbriata* supplementation therefore it is unlikely to recommend *C. fimbriata* as a weight loss supplement and an appetite suppressant.

**Supplementary Information:**

The online version contains supplementary material available at 10.1186/s12906-021-03450-8.

## Background

Obesity prevalence has increased during the past few decades, causing a pandemic. The World Health Organisation (WHO) global estimates in 2016 showed that more than 1.9 billion adults (≥18 years) were overweight, out of which 650 million were obese [1]. In the last three decades, the global prevalence of obesity has increased 27.5%, with many factors such as consumption patterns, urban developments and lifestyle habits having an influence on this increase in prevalence [2]. This exponential increase has parallelly caused an influx of other medical co-morbid conditions including; diabetes, stroke, cardiovascular disease, hyperlipidaemia, cancers, nonalcoholic fatty liver disease, polycystic ovarian syndrome and osteoarthritis [3]. In an obesogenic environment many factors influences weight gain, these include; positive energy balance due to increased energy intake compared to expenditure and appetite. Appetite forms a connective bridge between the internal and external environments, and may influence obesity by increasing the desire to eat, inappropriate food choices or the weak inhibition of eating [4]. Strategies for treating obesity, primarily involve lifestyle interventions for changes in diet and physical activity. Pharmocotherapy, is considered as a second line treatment, often recommended when lifestyle modifications are ineffective [5].

Currently there are no safe and effective pharmaceutical agents to reduce appetite and promote weight loss. Sibutramine and Rimonabant were approved appetite supplements and anti-obesity drugs, which were subsequently withdrawn due to serious cardiovascular and psychiatric adverse effects respectively [6, 7]. This has resulted in the focus on weight reduction by appetite suppresents shifting towards herbal products [8]. Plant extracts have been used for centuries in the Eastern world, however only recently has it gained wide popularity in western medicine for preventing diet induced obesity and reduction of body weight [5]. Many studies around the world has evaluated numerous herbal ingredients for weight-loss. One such study which looked at weight loss products in the Sri Lankan market reported that top ingredients used were herbal in origin: *Camellia sinensis*, *Garcinia cambogia*, *Cinnamomum zeylanicum* and *Zingiber officinale* [9].

*Caralluma adscendens var. fimbriata* (Wall.) Gravely & Mayur, is a plant that has generated an increased interest among researchers, commonly known as *Caralluma fimbriata* (*C.fimbriata)* is well spread in the dry regions of Asia and the genus previously belonged in the Asclepiadaceae family but at present has been merged into the Apocynaceae family under the subfamily Asclepiadoideae [10]. It is an edible succulent cacti which is a well known famine food, appetite suppressent, thirst quencher and grows wild all over India [11]. Claims in North Indian forklore suggests that this specific cacti consists of appetite supressent activity. Standardised extracts of *C. fimbriata* have been approved as safe and effective therapy for managemnent of obesity and overweight in Australia, India and USA [12]. Key phytochemical constituents of this plant includes pregnane glycosides, flavone glycosides, megastigmane glycosides and saponins [13]. Many in-vivo animal and human studies have investigated its effects on appetite and weight reduction. For example one study among Wistar rats with high fat diet induced insulin resistance, showed that the plant extract possess anti-hyperglycaemic, insulin sensitive, lipid lowering and antioxidant properties [14]. A study conducted by Kuriyan et al. in humans using an extract made from the aerial parts of the plant suggested that *C. fimbriata* has appetite suppressing action, where hunger levels were reduced with individuals reporting a feeling of fullness, with associated reduction in waist circumference (WC) [11]. Such positive clinical results proposes that *C. fimbriata* could possibly be used as an over-the-counter appetite supressant. However a single study does not usually present conclusive evidence on efficacy and saftety. It is important to systematically review the scientific literature to further identify if *C. fimbriata* could be used as an anti-obesity drug in the clinical practice. Therefore, the present study was conducted with the aim to systematically evaluate the literature, identify, compare and contrast the studies focusing on *C. fimbriata* and its effect on appetite supression and changes in obesity related anthropometric and biochemical parameters.

## Methods

A systematic review of clinical trials reporting the effects of *Caralluma fimbriata* as appetite suppression and anti-obesity supplement was reported in accordance with the Preferred Reporting Items for Systematic Reviews and Meta-Analyses (PRISMA) guidelines and PRISMA check list is attached as supplementary file 1 [15]. There is no review protocol present for this study.

### Search strategy

A comprehensive search of the literature was conducted in three databases: PubMed® (U.S. National Library of Medicine, USA), Web of Science® (Thomson Reuters, USA) and SciVerse Scopus® (Elsevier Properties S. A, USA) for studies published until 30th April 2020. The details of search key words are shown in supplementary Table 1. No language limit was applied and non-English manuscript were translated by Google translater. Moreover, no age restriction was used, while editorials, conference proceedings, commentaries and book reviews/chapters were excluded. The reference lists of the articles selected were screened manually in order to obtain additional data. Subsequently, total hits obtained from the three databases were pooled together and duplicate articles were removed. Retrieved articles were screened by reading the article ‘title’, ‘abstract’ and ‘full-text’. The filtered articles were further screened by reading the individual manuscripts, and those not satisfying inclusion criteria (given below) were excluded. This search process was executed by two independent reviewers (RJ, TVF) and the final group of articles incorporated in the systematic review was determined after an iterative consensus process.

### Inclusion and exclusion criteria

A study was considered eligible for data analysis if it met the criteria established by PICO index, a framework to formulate or deconstruct the research question: (1) Population: individuals with obesity or overweight with a BMI cut off of 25 kg/m^2^ and greater or syndromes that results from either being overweight or obese; (2) Intervention: supplementation with *C. fimbriata* with or without other substances; (3) Comparison: receiving placebo or no treatments; (4) Outcome: obesity anthropometric parameters (Body Mass Index (BMI), Body weight (BW), WC, Hip Circumference (HC), Waist to Hip ratio (WHR), body fat percentage (%), biochemical parameters (Lipid profile and Fasting blood Glucose (FBG)) and/or appetitie related parameters (Virtual Analogue Scale (VAS) or Hyperphagia Qusestionnaire).

### Data extraction, quality assessment and statistical analysis

Data extraction was done for all included studies by one researcher (RJ) and cross-checked by another (TVF). The following information was extracted from each study: 1) details of the study (first author, country, year of publication), 2) study design and duration of it, 3) study population, 4) details of the study sample, 5) details of the supplement and placebo, 6) both obesity related parameters including metabolic parameters, 7) appetite and nutritional details, 8) significant outcomes were evaluated. Any discrepancies in the data extracted in this manner were rechecked and resolved by discussion with the third reviewer (PR). The quality of studies were also assessed independently by two authors according to the Jadad scale (RJ, TVF). The three point based scale is used to evaluate the clinical studies that have been utilised in this review.

Meta-analysis of outcomes were performed using Review Manager Software, version 5.3. Where available, the mean and standard deviation (SD) for baseline, end, and change from baseline values, as well as mean differences within or between intervention and comparison arms, were extracted for each outcome. Missing standard deviations were calculated from confidence intervals, standard error, or *P* values for difference in means following Cochrane guide. When these data were unavailable, standard deviations were imputed using a pooled correlation coefficient derived from a meta-analysis of correlation coefficients from studies reporting sufficient data. When there is not enough information available to calculate the standard deviations for the changes, they can be imputed as per guidelines provided in the Cochrane handbook [16]. When significant heterogeneity was noted in the pooled outcomes (I^2^ > 60%) a random-effects model was used for meta-analysis, otherwise a fixed-effects model was employed. In all analyses, a *p* value < 0.05 was considered statistically significant.

## Results

### Search results

**Fig. 1:**
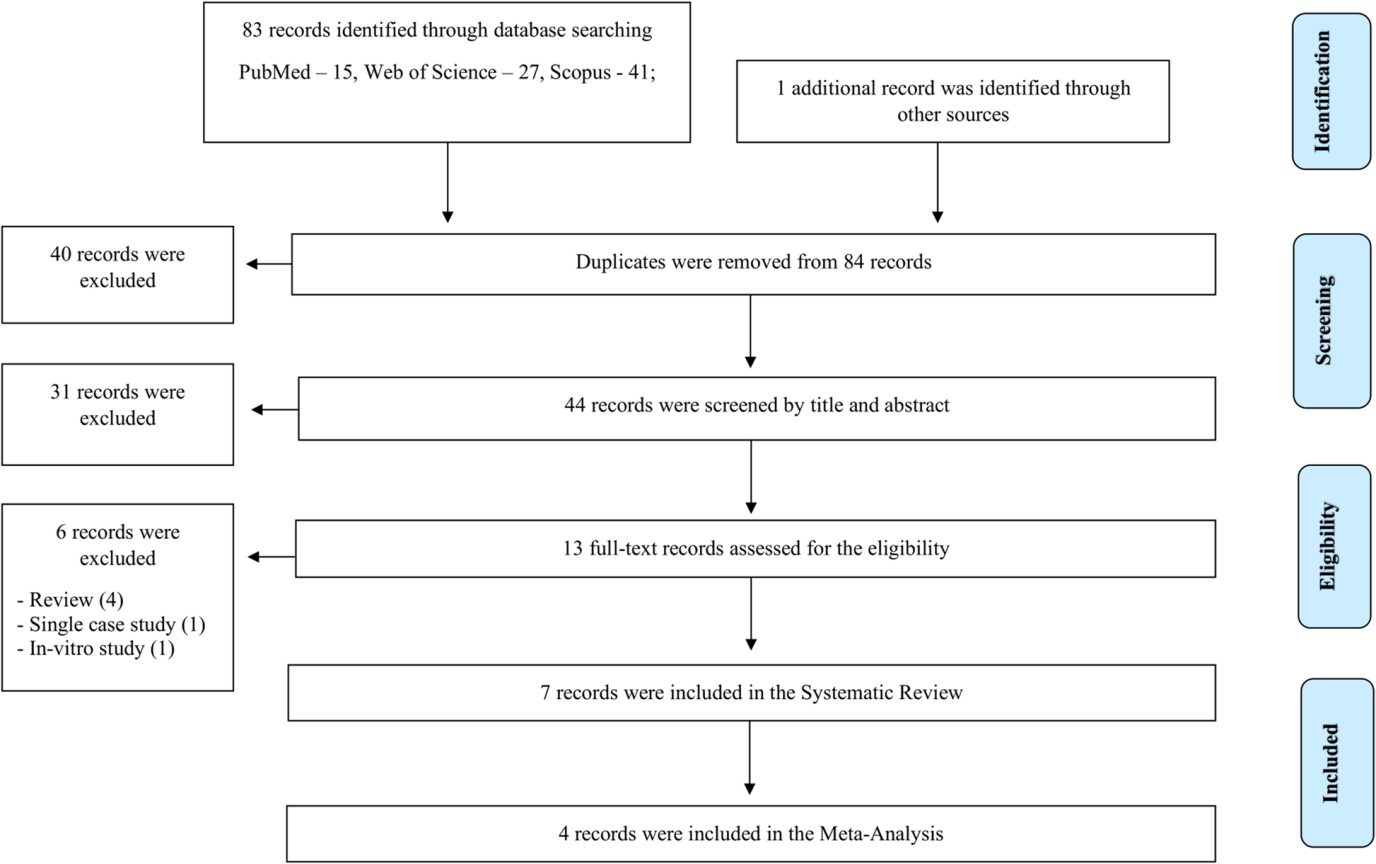
Summarised search strategy

A total of 83 articles were retrieved by searching the respective database; PubMed (15), Web of science (27) and Scopus (41). One additional article was identified by other sources. After removing duplicates 44 were selected for the title screening. Thirteen articles were included after screening by title and abstract. Finally, seven papers satisfied the inclusion/exclusion criteria and were included to the systematic review. One study was reported in Spanish, while all others were in English. A detail of the search strategy is summarised in Fig. 1 (PRISMA flow chart).

### Description of studies

The data on effect of *C. fimbriata* as an appetite suppressant and an anti-obesity treatment of various studies were extracted, which originated from four different countries: Australia (3), Cuba (1), India (2) and Spain (1). Study design of most studies included were randomised, double blinded and placebo controlled (*n* = 4) [5, 11, 17, 18]. A difference in study design was observed among three studies. Two studies conducted randomisation but with one study double blinded and the other placebo controlled. (*n* = 2) [19, 20]. The remaining third study was an open labeled study [21] (Table 1). According to the Jadad scale, five studies out of the seven studies obtained a score greater than 3 showcasing the strength of the study, it met the main three criterias; randomisation, blinding and the total account of the participants in the study individually. Almost all studies recruited adults who were overweight or obese with a BMI > 25 kg/m^2^ (*n* = 5) and one other study recruited healthy adults only with a BMI average of 26.5 kg/m^2^. Of the two studies that recruited overweight and obese individuals, one additionally screened for central adiposity by using waist circumference and the other assessed individuals with both impaired fasting glucose (IFG) and normal glucose levels. One study recruited children and adolescents with Prader-Willi syndrome (PWS) to assess their appetite behaviours. As seen from all the studies in Table 1, the range of sample size of the studies reviewed were of a minimum of 15 to maximum of 97. Age range of individuals were from 9 to 70 years and only one study focused solely on females whereas all other studies were both genders (*n* = 6). Any side effects of individuals who ingested *C. fimbriata* were reported by few studies*,* of which most common effects were; constipation, diarrhoea, nausea and rashes (*n* = 4).

**Table 1:** Summary of included clinical studies

Authors^**[ref]**^; Year of Publication; Country	Study Design; Duration; Jadad Score	Study population	Sample Size (I / C); gender; Age	CFE formulation and dose; Placebo	Parameter(s) Studied			Significant Outcomes		Adverse side effects
					Obesity (Ob) Metabolic (M)		Appetite	Obesity/ Metabolic	Appetite	
Arora et al. [19]; 2015; India	R, PC; 12 weeks; 4 points	Overweight or obese BMI > 25 kg/m^2^	89 (47 / 42); Both genders; 18–50 years	CFE capsule 0.5 g/bd; Placebo	Ob M	BW, BMI, WC, HC, WHR, TC, HDL, LDL, HDL: LDL, VLDL, FBS, PPS, ALS, ALT, ALP, RFT, CBC, BP, HR, ECG	VAS appetite assessment: hunger, thoughts of food, urge to eat, fullness of stomach	NS	NS	Nausea (8.5%), Palpitation, Glossitis, Insomnia (4.2%), Generalized weakness (10.6%), Constipation, Exacerbation of blood pressure (2.1%)
Astell et al. [5]; 2013; Australia	R, DB, PC; 12 weeks; 5 points	Overweight or centrally obese BMI > 25 kg/m^2^ or WC > 94 cm (male), > 80 cm (female)	33 (17 / 16); Both genders; 29–59 years	CFE capsule 0.5 g/bd; Placebo	Ob M	BW, BMI, WC, HC, WHR, SBP, DBP, HR, FBS, TAG, TC, HDL, LDL, HDL: LDL, Leptin	Energy and nutrient intake, VAS appetite assessment: hunger, desire to eat, fullness of stomach	WC, WHR	Palatability (visual appeal, smell, taste) sodium intake	Mild rash and constipation earlier on and then subsided
Cabrera-Rode [20]; 2017; Cuba	OL; 3 months; 1 point	Overweight and obese with/without IFG BMI = 25–44 kg/m^2^	40 (20 IFG, 20 non-IFG); Both genders; 23–60 years	Obex powder sachet [1.5 g CFE] bd, before main meal	Ob M	BW, BMI, WC, HC, WHR, WhtR, SBP, DBP, FBG, FI, HbA1c, TC, TAG, HDL, Creatinine, UA, ALT, AST, GGT, Hb, Serum Iron, HOMA-β, HOMA-IR, QUICKI, Bennett, Raynaud	NR	WT, BMI, WC, WHR, WHtR, HC (+IFG), BP (−IFG), FBS (+IFG), IF (+IFG), HDL, HOMA-β(+IFG), HOMA-IR (+IFG), QUICKI(+IFG), Bennett (+IFG), Raynaud (+IFG)	NR	Rashes, headache, diarrhoea, nausea, dyspepsia and bloating were recorded
Griggs et al. [16]; 2015; Australia	R, DB, PC, CO; 4 weeks; 4 points	Prader-willi Syndrome, NG	15; Both genders; 9.27 ± 3.16 years	CFE capsule 1.0–0.5 g (250 mg / 10 kg BW); Placebo	Ob	BW	Hyperphagia questionnaire	NR	Hyperphagia	NR
Kell et al. [17]; 2019; Australia	R, DB, PC; 8 weeks; 5 points	Healthy –overweight BMI < 30	97 (49 / 48); Both genders; 18–70 years	CFE capsule 0.5 g/bd; Placebo	Ob	BW, BMI		NS	NG	NG
Kuriyan et al. [10]; 2007; India	R, DB, PC; 60 days; 5 points	Overweight or obese BMI > 25 kg/m2	50 (25 / 25); Both genders; 25–60 years	CFE capsule 1 g/day; Placebo	Ob M	BW, BMI, WC, HC, fat% FBS, PPS, TC, HDL, LDL, TAG	Energy and nutrient intake, VAS appetite assessment: hunger, thoughts of food, urge to eat, fullness of stomach	WC	Hunger (%)	abdominal distention, flatulence, constipation and gastritis (24%)
Laura et al. [15]; 2015; Spain	R, DB; 2 months; 1 point	Overweight or obese BMI > 25 kg/m2	44; Woman; 35–62 years	Obex powder sachet [1.5 g CFE] bd, before lunch and dinner	Ob M	BW, Bicipital fold, Triceps fold, Subscapular fold, suprailiac fold, %fat, Total muscle mass, arm muscle mass, leg muscle mass, trunk muscle mass, body water%, SBP, DBP, FBS, TC	NG	Bicipital fold, Triceps fold, Subscapular fold, suprailiac fold, trunk muscle mass, %	NG	NG

*ALS* Amyotrophic lateral sclerosis; *ALT* Alanine transaminase; *ALP* Alkaline phosphate; *AST* Aspartate Aminotransferase; *B* Biochemical; *BMI* Body mass index; *BW* Body weight; *BP* Blood pressure; *CBC* Complete blood count; *CFE Caralluma Fimbriata* Extract; *CO* Crossover; *DB* Double blind; *DBP* Diastolic blood pressure; *ECG* Electrocardiography; *F1* Fibrosis; *FBG* Fasting blood glucose; *GGT* Gamma glutamyl transferase; *Hb* Haemoglobin; *HbA*_*1C*_ Glycated haemoglobin; *HC* Hip circumference; *HDL* High density lipoprotein; *HOMA* IR – Homeostatic model assessment – Insulin resistance; *HOMA – β* Homeostatic model assessment – Beta cell function; *HR* Heart rate; *IFG* Impaired fasting glucose; *LDL* Low density lipoprotein; *M* Metabolic; *NR* Not reported; *NG* Not given; *NS* Not shown; *Ob* Obesity; *OL* Open labeled; *PC* Placebo controlled; *PPS* Postprandial sugar; *QUICKI* Quantitative insulin sensitivity check index; *R* Randomised; *RFT* Renal function test; *SBP* Systolic blood pressure; *TC* Total cholesterol; *TAG* Triglycerides; *UA* Urinalysis; *VAS* Visual analogue scales; *VLDL* Very low-density lipoprotein; *WC* Waist circumference; *WHR* Waist to hip ratio; *WHtR* Waist to height ratio

### Effect of *C. fimbriata* for obesity

**Fig. 2:**
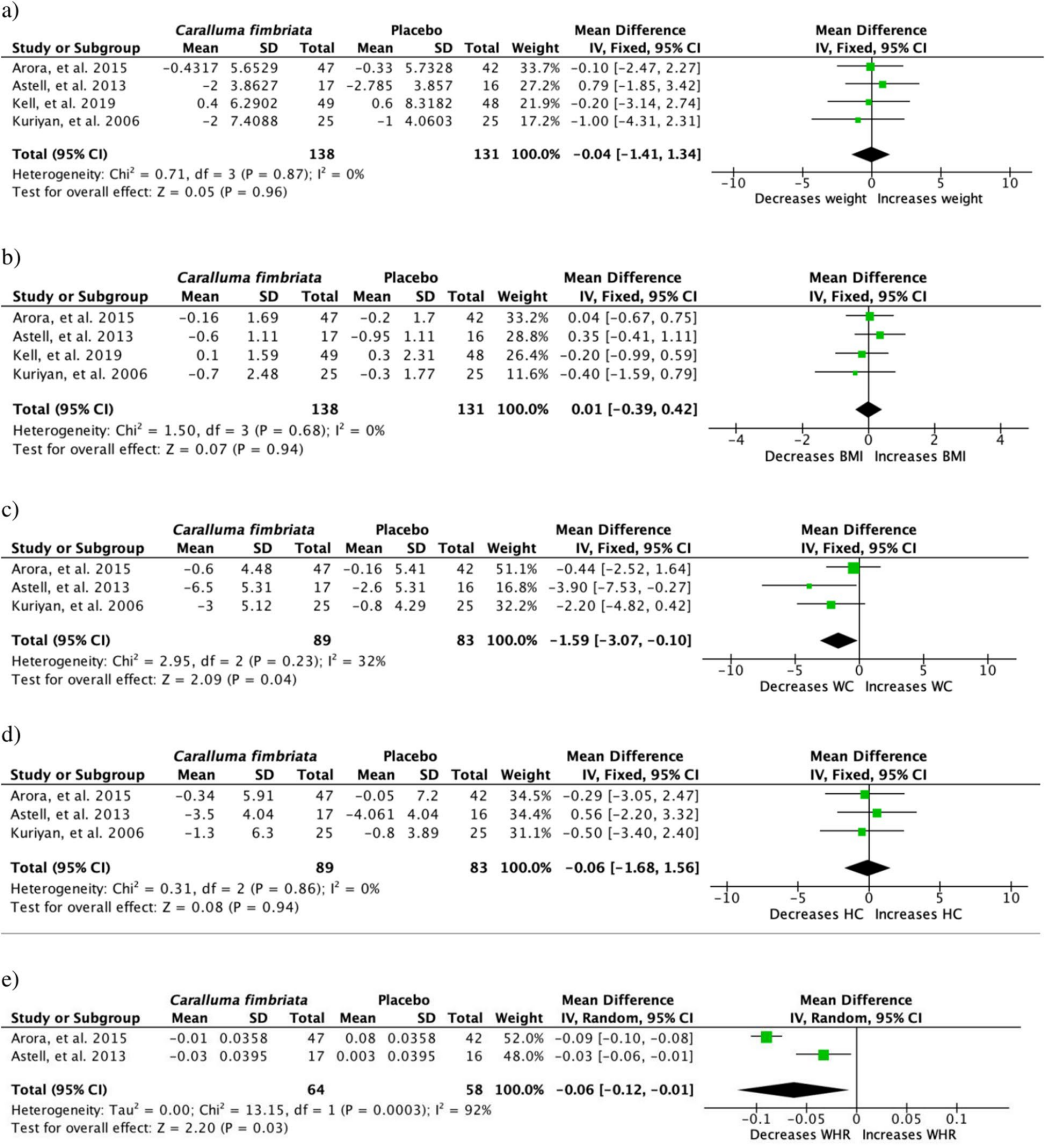
Clinical trials studying effect of *Caralluma fimbriata* on anthropometric parameters **a** body weight [BW], **b** body mass index [BMI], **c** waist circumference [WC], **d** hip circumference [HC] and **e** waist to hip ration [WHR]

Several parameters used to assess obesity were collected. Anthropometric parameters as BW were measured in all the studies reviewed whereas most studies included the following parameters in addition; BMI, WC, HC, WHR (*n* = 5). Only certain studies measured body fat percentage (*n* = 2) and another study measured skin fold thickness, muscle mass and body water percentage. As shown in Fig. 2 four studies were included in the meta-analysis. The four studies included in the analysis all included a placebo controlled group. Compared to placebo controlled group, *C. fimbriata* extract significantly reduced WC by − 1.59 cm (95% CI, − 3.07, − 0.10, *p* = 0.041; I^2^ = 32%, *p* = 0.23) and WHR by − 0.06 (95% CI, − 0.12, − 0.01, *p* = 0.05; I^2^ = 92%, *p* = 0.0003) respectively. However, no significant effects were seen on BW, BMI and HC.

### Effect of *C. fimbriata* for associated biochemical parameters

**Fig. 3:**
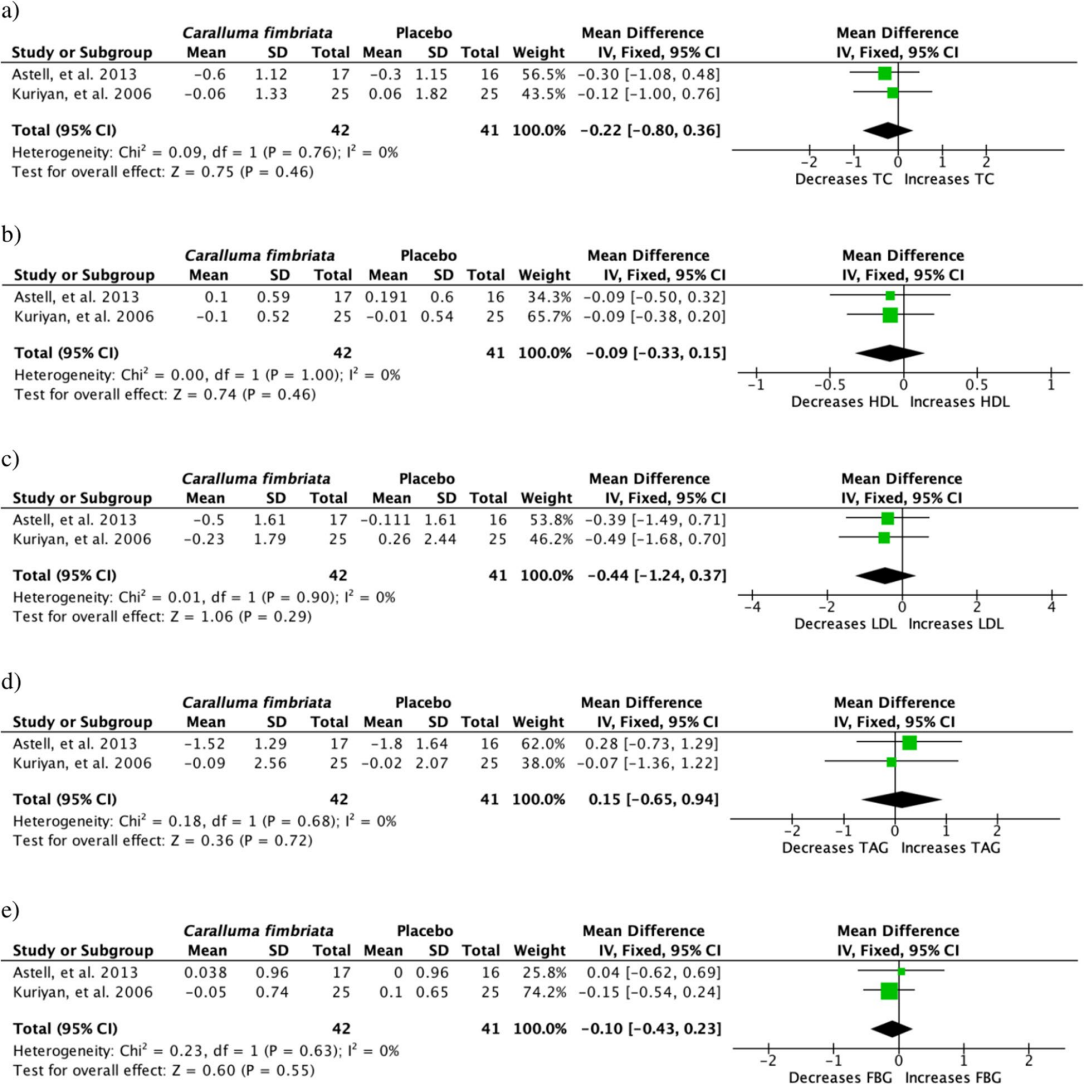
Clinical trials studying effect of *Caralluma fimbriata* on biochemical parameters **a** total cholesterol [TC], **b** HDL cholesterol, **c** LDL cholesterol, **d** triglycerides [TAG] and **e** fasting blood glucose [FBG]

Biochemical parameters assessed in studies covered were of multiple tests as lipid profile (Total Cholesterol (TC), High Density Lipoprotein (HDL), Low Density Lipoprotein (LDL), Triacylglycerides (TAG)) and FBG levels (*n* = 4). Table 1 shows other tests that were carried out by the other studies with one standing out as it assessed levels of appetite hormone leptin (1). Aforementioned biochemical parameters were included in the meta-analysis and outcome shows that *C. fimbriata* had no significant changes (*P* > 0.05) on the biochemical parameters analysed (Fig. 3).

### Effect of *C. fimbriata* for appetite

**Fig. 4:**
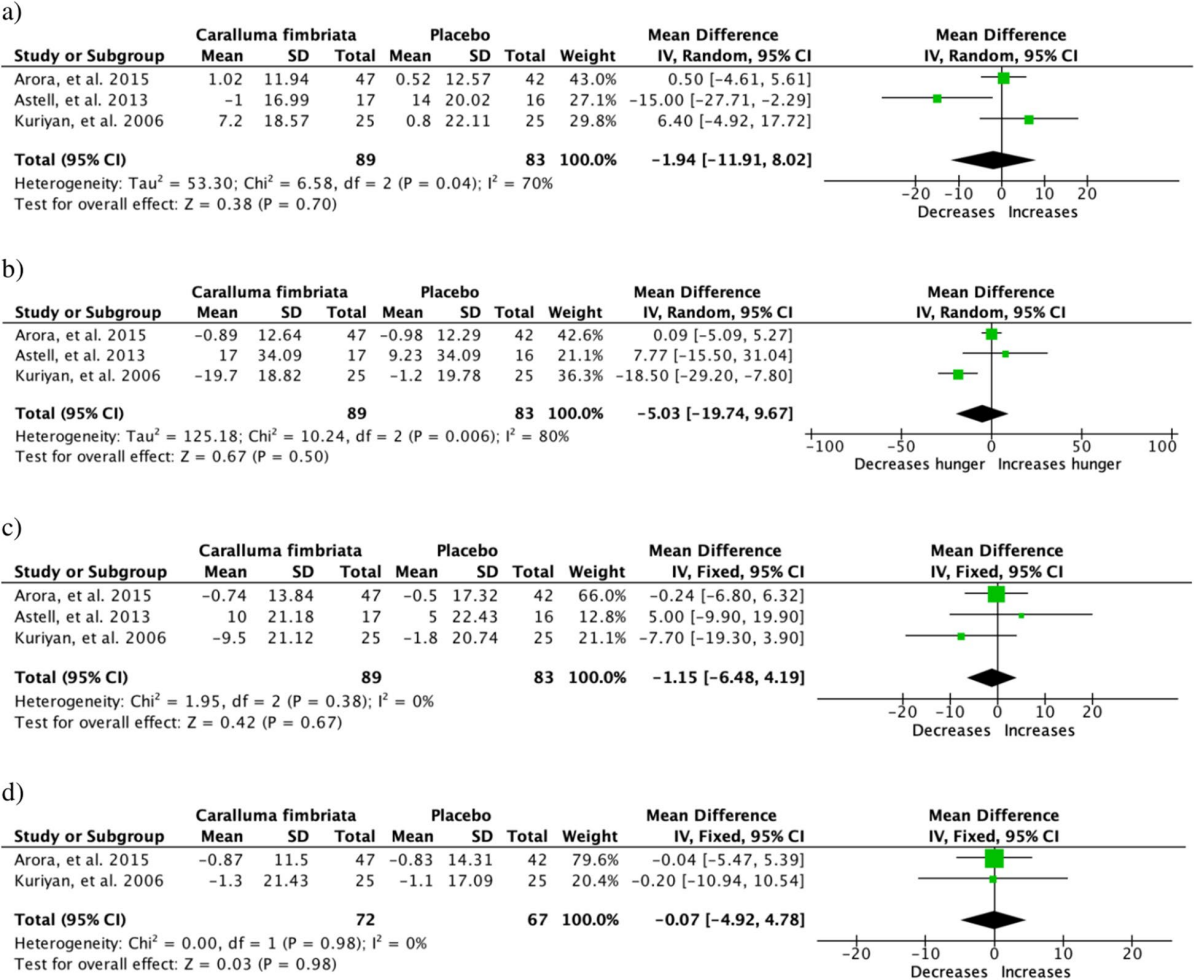
Clinical trials studying effect of *Caralluma fimbriata* on appetite **a** feeling of fullness, **b** hunger, **c** desire to eat and **d** thoughts on food

Appetite suppressing characteristic of *C. fimbriata* was assessed by few studies by either utilising VAS (*n* = 3). or the Hyperphagia questionnaire (*n* = 1). VAS has four components to its assessment which consists of: hunger, thoughts of food, fullness of stomach and urge to eat. Three studies were equipped with VAS, out of which the studies that assessed all four aspects of the VAS assessment (*n* = 2) the study by Kuriyan et al. showed the only component of the scale that had effect was hunger as the experimental group found a 19.7% decrease in hunger post intervention. The study that utilised the Hyperphagia questionnaire including of 13 questions was specifically validated for PWS. This questionnaire allowed evidence for any change in satiety or behaviour to be marked with a five step gradient multiple choice range and a significant accumulative hyperphagia decrease (*P* = 0.009) was observed with the individuals who ingested the higher dose. The results of the meta-analysis showed no significant effect to the any of appetite parameters (Fig. 4).

## Discussion

This is the first systematic review and meta-analysis as far as we know evaluating the efficacy of *C. fimbriata* as a weight reduction supplement and appetite suppressant towards anti-obesity treatment. Pre-clinical studies on rat models reported considerable beneficial effects of *C. fimbriata* as anti-obesogenic [22], appetite suppressing [23] and antihyperglycemic [24] agent. However, this meta-analysis revealed such characteristics of *C. fimbriata* were found to be ineffective in reducing body weight in obese or overweight individuals, although a reduction was seen in WC. Furthermore, it did not show promising effects in controlling appetite as well, with no positive effects on biochemical parameters such as lipid profile and plasma blood glucose. Many herbal products have been used in weight reduction, as it is anticipated that the availability of many natural sources is considered as a supporting tool to keep obese people holding onto their weight loss [25]. Systematic reviews studying certain specific ingredients showed mean weight loss effects of *Garcinia cambogia*, *coffea canephora robusta, phaseolus vulgaris* which are 0.88 kg (95%CI: − 4.23, − 0.72; *P* = 0.05), 2.47 kg (95%CI: − 1.75, − 0.00; *P* = 0.006), 1.77 kg (95%CI: − 3.33, 0.33; *P* = 0.10) respectively in kilograms of body weight [26–28]. *C. fimbriata* reported virtually no weight loss comparatively to the other extracts which is − 0.04 (− 1.41,1.34; *P* = 0.96) in kilograms only.

Additionally, certain animal studies have been carried out to examine the potential of *C. fimbriata* as being not only anti-obesogenic [22] but having an effect on insulin resistance and oxidative stress in rat models [29]. A study using diet induced obesity in Wistar rats reported an improvement in lipid profile with *C. fimbriata* supplementation [30]. Another study working with diet induced rat models to study anorexigenic and obesogenic properties resulted in *C. fimbriata* effects to bring about dose dependent changes in both properties [22]. A mechanism on appetite was further investigated reinforcing the effect of *C. fimbriata* extract on food intake in the Snord116del mouse which involved 5-HT2cR and identified this 5-HT2cR played a role in induced appetite suppression and significant stimulatory feeding disruptions in this mouse model [31]. Nonetheless, experimenting on rat models with *C. fimbriata* showed many promising results for its work as an anti-obesogenic treatment but were not replicated in the human studies. Appetite suppressing characteristics have been assessed by few studies using the VAS and one study using the Hyperphagia questionnaire as both tools study more on the individual’s appetite behaviors further affecting food intake. All studies analysed in the meta-analysis for appetite suppressant behaviors showed no significant changes with *C. fimbriata* ingestion. However, assessing one’s perception post ingestion of the extract utilising either a scale or a questionnaire would not be sufficient in concluding the effect of the *C. fimbriata* extract as an appetite suppressant. It would strengthen the effects if testing for appetite hormones were included as biological mechanisms of appetite and satiety, as they are regulated by a complex interaction between neurological and hormonal signals therefore increasing the understanding of the effect of *C. fimbriata* [32].

A toxicology assessment to evaluate the safety of a hydroethanolic extract of *C. fimbriata* was conducted; a compilation of toxicological tests, a 6 month oral toxicity study and a developmental toxicity in rats concluded that oral consumption of the *C. fimbriata* extract was safe [33]. However, four out of seven studies have reported mild to modest adverse reactions of *C. fimbriata*, of which few common effects as reported were constipation, diarrhea and rashes.

This systematic review has several limitations. Studies were searched across the three key databases but not the ayurvedic database which may have included more studies exploring *C.fimbriata*. In addition, other studies published in non-indexed journals were not included and that may have presented important information. The studies included mention that the aerial parts of the plant were used for the *C.fimbriata* extract but do not indicate if it was the flower, leaves or stem etc. In addition, the process of extraction and the solvent used is not mentioned consistently across the studies. Two studies reviewed the usage of the dietary supplement Obex which resulted in significant reduction of most anthropometric parameters, which is made out of a mixture of active ingredients including *C. fimbriata* and other ingredients [21] thus combining studies analysing on both Obex and *C. fimbriata* extract. Obex is a dietary supplement powder sachet whereas the other studies utilised *C.fimbriata* extract in the form of capsules. Another limitation is that from the seven studies, only four were able to be utilised for the meta-analysis as due to lack of placebo controlled group in the other studies although the quality of included studies was of 4–5 Jadad score. A funnel plot asymmetry was not tested as less than 10 studies were included and if tested it would be difficult to distinguish biasness. Appetite suppressant effects were analysed from the studies, but energy and macronutrient intake affected by the results of suppressed appetite was not incorporated into the meta-analysis. These factors prevent us from drawing a meaningful conclusion about the potential impact to food intake of *C. fimbriata* to farther use it as an obesity treatment option.

## Conclusion

This review reported seven interventional studies on supplementation of *C. fimbriata* for weight reduction and/or appetite suppression. Meta-analysis of four placebo controlled studies shows no significant reduction on BW, BMI, HC except WC and WHR. Appetite parameters: thoughts of food, urge to eat, fullness of stomach showed no significant changes except for hunger. No metabolic parameters improved with *C. fimbriata* supplementation and mild to moderate adverse effects were commonly reported. According to our research, it is difficult to recommend *C. fimbriata* as a weight loss supplement and an appetite suppressant.

## Supplementary Information

**Additional file 1.**

**Additional file 2.**

## Data Availability

The datasets used and/or analysed during the current study available from the corresponding author on reasonable request**.**

## Abbreviations

BW: Body weight
BMI: Body mass index
WC: Waist circumference DM diabetes mellitus
FPG: Fasting plasma glucose
HbA1c: Glycosylated hemoglobin
HDL: High-density lipoprotein
LDL: Low-density lipoprotein
MI: Myocardial infarction
TC: Total cholesterol
TG: Triglycerides
