# Supplementary material for: The use of Caralluma fimbriata as an appetite suppressant and weight loss supplement: a systematic review and meta-analysis of clinical trials
Source: BMC Complement Med Ther. 2021 Nov 10;21:279. doi: 10.1186/s12906-021-03450-8 (PMC8579607; doi:10.1186/s12906-021-03450-8)
Supplement: Supplementary file 1 — Additional file 1. [file 12906_2021_3450_MOESM1_ESM.docx]

83 records identified through database searching

PubMed – 15, Web of Science – 27, Scopus - 41;

## **Screening**

## **Included**

## **Eligibility**

## **Identification**

44 records were screened by title and abstract

40 records were excluded

13 full-text records assessed for the eligibility

7 records were included in the Systematic Review

1 additional record was identified through other sources

Duplicates were removed from 84 records

31 records were excluded

6 records were excluded

- Reviews (4 records)

- Single case study (1 record)

- In-vitro study (1 record)

4 records were included in the Meta-Analysis

**Figure 4:** Summarized search strategy
