# Supplementary material for: The use of Caralluma fimbriata as an appetite suppressant and weight loss supplement: a systematic review and meta-analysis of clinical trials
Source: BMC Complement Med Ther. 2021 Nov 10;21:279. doi: 10.1186/s12906-021-03450-8 (PMC8579607; doi:10.1186/s12906-021-03450-8)
Supplement: Supplementary file 2 — Additional file 2. [file 12906_2021_3450_MOESM2_ESM.docx]

**Supplementary Table 1:** Search strategy

| 1 # (slimaluma OR caralluma OR "Caralluma fimbriata" OR "Caralluma R.Br.") |
| --- |
| 2 # (Obesity OR overweight OR “weight reduction” OR “body fat” OR appetite) |
| 3# 1# AND 2# |
| All age, all languages  Excluded: conference proceedings, commentaries, editorials and book reviews/ chapters  Date: until 30^th^ April 2020 |
